# Colonoscopic removal of an intrauterine device with rectal perforation: A case report

**DOI:** 10.1097/MD.0000000000038872

**Published:** 2024-07-12

**Authors:** Lingrun Ye, Yuanyuan Zhu, Fanglai Zhu

**Affiliations:** a Gastroenterology Department, Wannan Medical College, Wuhu, Anhui, China; b Department of Gastroenterology, Anqing First People’s Hospital Affiliated to Anhui Medical University, Anqing, Anhui, China.

**Keywords:** endoscopic treatment, intrauterine device, rectum

## Abstract

**Rationale:**

The intrauterine device is one of the effective, safe, convenient, economical, and reversible contraceptive methods. Although its contraceptive effect is definite, some female patients may experience complications such as expulsion, bleeding, and pregnancy with the device in place. Rectal perforation is one of the rare and serious complications, which can lead to complications such as abdominal infection and intestinal adhesions, severely affecting the quality of life of patients.

**Patient concerns:**

A 34-year-old female was sent to the Department of Gastroenterology with noticeable left lower quadrant abdominal pain. She had presented with abdominal discomfort and anal tenesmus 1 year earlier. Two months ago, her abdominal pain had gradually worsened and she was presented to our hospital.

**Diagnoses:**

Investigations, including colonoscopy and computed tomography scan, had revealed an intrauterine device migrated and perforated into the rectum.

**Interventions and outcomes:**

The patient underwent successful colonoscopic removal of the intrauterine device. She recovered well after the treatment.

**Lessons:**

This case proves that endoscopic therapy can be considered the preferred method for removing intrauterine devices displaced into the digestive tract lumen.

## 1. Introduction

The intrauterine device (IUD) is one of the most widely used methods for reversible contraception with high effectiveness of more than 99%.^[1]^ However, it can cause rare but severe complications, including bleeding, abdominal pain, migration, and perforations.^[2]^ The incidence of complications is very low. The rate of uterine perforation ranges from 0.5 to 13 per 1000 individuals.^[3]^ And the rate of IUD migration is reported to be 0.1% to 0.9%.^[4]^ Of the cases of gastrointestinal perforation, rectal perforation is uncommon. This report describes a case of ectopic migration of an IUD with perforation of the rectum.

## 2. Case presentation

A 34-year-old patient had an IUD inserted following her first pregnancy. Despite the insertion, she got pregnant and delivered a child 5 years later. After giving birth to the second baby, she had another IUD inserted, without being aware that the first IUD had migrated. One year later, the patient experienced the symptoms of tenesmus, sagging sensation in the anus, and left lower quadrant abdominal discomfort. She ignored it and did not visit a hospital. However, 2 months ago, her abdominal pain had gradually worsened and she was presented to our hospital.

## 3. Investigations

**Figure 1.**
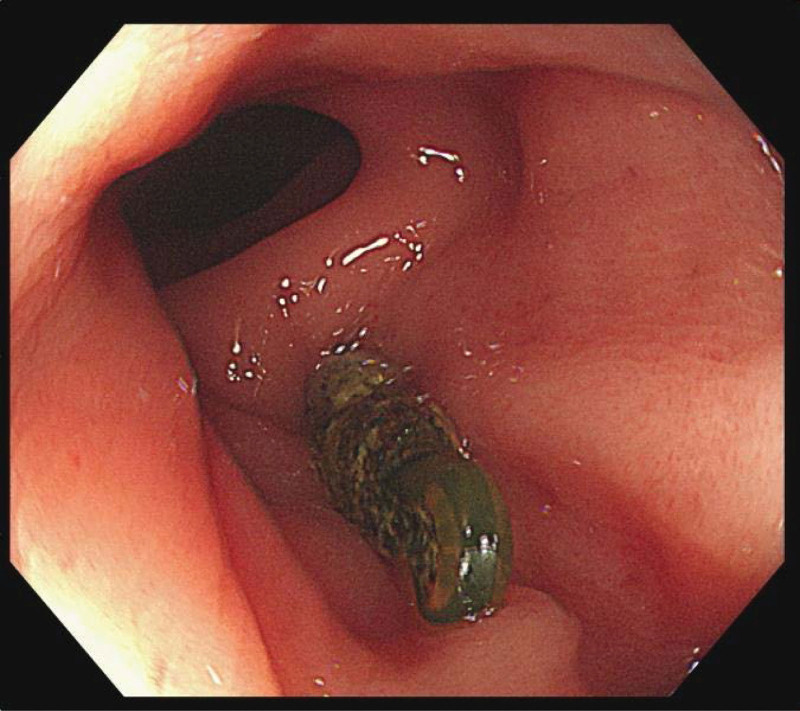
Colonoscopy revealed an IUD perforating the rectum 10 cm from the anal verge. IUD = intrauterine device.

**Figure 2.**
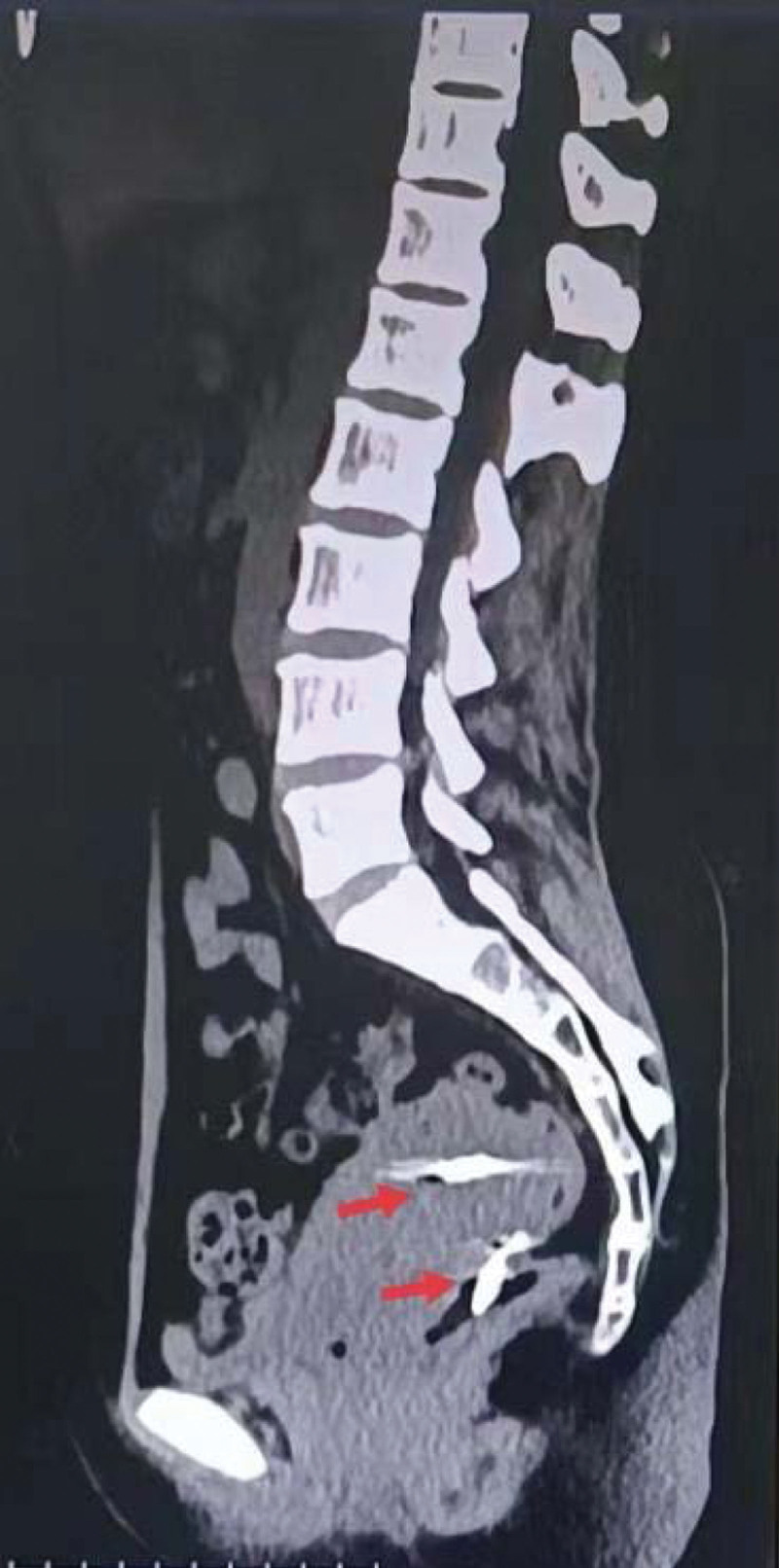
CT images of 2 intrauterine devices. One was intrauterine (indicated by the red arrow at the top), and the other penetrated into the intestinal cavity (indicated by the red arrow at the bottom). CT = computed tomography.

Colonoscopy revealed an IUD that had perforated the rectum 10 cm from the anal verge (Fig. 1). Abdominal computed tomography demonstrated 2 IUDs: one intrauterine and the other outside the cavity. One end of the second IUD was found in the abdominal cavity, and the other was perforated into the rectal wall (Fig. 2).

## 4. Treatment

**Figure 3.**
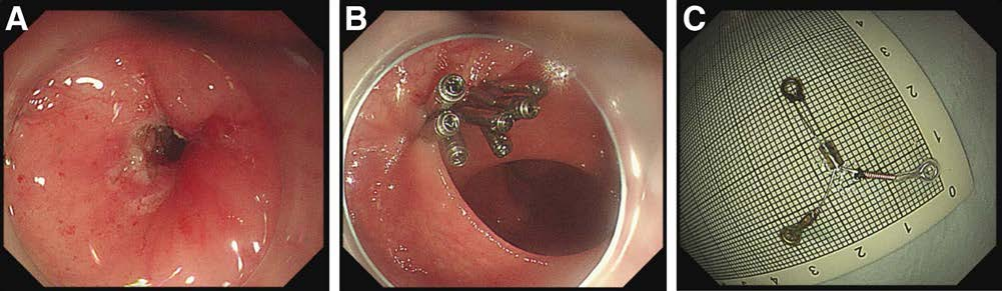
Colonoscopic retrieval of the IUD. (A) The wound site after the IUD had been removed. (B) The titanium clips at the wound site. (C) The image of the retrieved intrauterine device. IUD = intrauterine device.

Under conscious sedation with propofol, a colonoscopy (260J; Olympus Optical, Tokyo, Japan) with a transparent hood was performed. Initially, intestinal irrigation was repeatedly performed with tinidazole solution. Then, the dental floss was fixed on one end of the IUD with a titanium clip. The rectal mucosa was incised using a hot oblique-oriented knife in the direction of penetration from the abdominal cavity to the rectal lumen. After that, we extended the incision by using an insulated-tip knife, and the IUD was freed and extracted from the anus using a foreign body clamp. The wound was sutured using 8 titanium clamps (Fig. 3A‐C). The patient received postoperative antibiotic therapy. On postoperative day 1, the patient had the normal passage of gas and low-grade fever with a body temperature of 37.7°C. A liquid diet was administered on postoperative day 2 and her body temperature returned to normal. She was discharged on postoperative day 4.

## 5. Discussion

An IUD is a long-acting, easily reversible, and safe female contraceptive method which is commonly used worldwide.^[5]^ However, for 10% of IUD-users, IUD needs to be removed due to complications.^[6]^ These complications include bleeding, expulsion, and perforation into the uterus, gastrointestinal tract, and urinary tract.^[7,8]^ Several risk factors such as: inexperienced medical staff, inappropriate time of the IUD implantation, and lack of follow up are reported to be correlated with the complications.^[9]^ Trained doctors and nurses have a higher rate of performing successful insertions. Expulsion is more likely to occur after immediate and early postpartum IUD implantation compared to interval implantation.^[10]^ Meanwhile, the timing of IUD placement is considered to be positively linked to migration.^[11]^ The possible mechanism of IUD migration to the gastrointestinal tract is speculated as 3 stages. Initially, the IUD moved from the uterus to the abdominal cavity along the uterine wall. After that, it migrated to the gastric serosa or intestinal serosa under the influence of visceral motility and inflammation; and then penetrated the gastrointestinal tract wall. Eventually, the IUD moved to the gastric cavity or rectal lumen. In the early stage of the postpartum period, the uterine wall is thin and soft, making it simple for IUD to be released from the uterus. Thus, the probability of expulsion is larger than that in other periods. While most perforations occur in the early stage or immediately after IUD implantation, in some unusual cases, they can occur several years later. Three phases are considered to constitute a possible colonic penetration process. Initially, the IUD migrated to the abdominal cavity. After that, IUD adhered to the pericolic fat. Then, due to the local inflammation, it penetrates the colon.

Imaging computed tomography scan, abdominal X-ray, and endoscopy are commonly used to diagnose migration and perforation. Treatment options for IUD removals can be endoscopy, laparoscopy, and laparotomy. Thanks to the advancement of endoscopic techniques, endoscopic removal has developed into safe and cost-effective management with less invasion, less post-procedure pain, short hospital stay, rapid postoperative recovery, and low complication rates. Because of patient preferences for minimally invasive treatments, endoscopic managements are more used to remove ectopic IUDs than before. However, the exact removal method must be determined based on IUD’s position and patients’ conditions. In particular conditions, endoscopy needs to be converted to laparotomy.

In our case, colonoscopy was used to successfully remove the migrated IUD perforated into the rectum. After retrospectively analyzing this case, it was suggested that the patient should have an interval IUD insertion and reexamine regularly after placement. When the IUD is missed in the uterine cavity, investigations should be performed to exclude the possibility of IUD migration.

## 6. Learning points

IUD perforation is uncommon, but suspicion should be raised in any patient with IUD missing in the uterine cavity. Preoperative investigations like colonoscopy and abdominal computed tomography are effective to confirm the location of the migrated IUD. Endoscopic treatment is a good way to treat intrauterine device displacement to the lumen of digestive tract.

## Author contributions

**Conceptualization:** Lingrun Ye, Fanglai Zhu.

**Formal analysis:** Yuanyuan Zhu.

**Funding acquisition:** Fanglai Zhu.

**Investigation:** Lingrun Ye.

**Methodology:** Lingrun Ye.

**Resources:** Fanglai Zhu, Yuanyuan Zhu.

**Supervision:** Fanglai Zhu, Yuanyuan Zhu.

**Writing – original draft:** Lingrun Ye.

**Writing – review & editing:** Fanglai Zhu.
